# Environmental Filtering Drives Fungal Phyllosphere Community in Regional Agricultural Landscapes

**DOI:** 10.3390/plants12030507

**Published:** 2023-01-22

**Authors:** Annika Hoffmann, Alexandra-Raluca Posirca, Simon Lewin, Gernot Verch, Carmen Büttner, Marina E. H. Müller

**Affiliations:** 1 Leibniz Centre for Agricultural Landscape Research (ZALF), 15374 Müncheberg, Germany; 2 Phytomedicine, Albrecht Daniel Thaer Institute, Faculty of Life Science, Humboldt-Universität zu Berlin, 10099 Berlin, Germany; 3 State Office for Rural Development, Agriculture and Land Reorganization (LELF) Brandenburg, Division P, 15236 Frankfurt (Oder), Germany

**Keywords:** regional filter processes, community assembly, ITS, LEfSe, *Fusarium*, wheat

## Abstract

To adapt to climate change, several agricultural strategies are currently being explored, including a shift in land use areas. Regional differences in microbiome composition and associated phytopathogens need to be considered. However, most empirical studies on differences in the crop microbiome focused on soil communities, with insufficient attention to the phyllosphere. In this study, we focused on wheat ears in three regions in northeastern Germany (Magdeburger Börde (MBB), Müncheberger Sander (MSA), Uckermärkisches Hügelland (UKH)) with different yield potentials, soil, and climatic conditions. To gain insight into the fungal community at different sites, we used a metabarcoding approach (ITS-NGS). Further, we examined the diversity and abundance of *Fusarium* and *Alternaria* using culture-dependent and culture-independent techniques. For each region, the prevalence of different orders rich in phytopathogenic fungi was determined: Sporidiobolales in MBB, Capnodiales and Pleosporales in MSA, and Hypocreales in UKH were identified as taxonomic biomarkers. Additionally, *F. graminearum* was found predominantly in UKH, whereas *F. poae* was more abundant in the other two regions. Environmental filters seem to be strong drivers of these differences, but we also discuss the possible effects of dispersal and interaction filters. Our results can guide shifting cultivation regions to be selected in the future concerning their phytopathogenic infection potential.

## 1. Introduction

Various agriculture strategies are currently being reviewed as part of the increased adaptation to climate change. In addition to agricultural diversification [1,2,3,4] and new ways to improve the water retention capacity of soils [5,6,7], the spatial (northern) shift of arable crop acreage is also discussed [8,9,10]. Even though shifting agricultural zones northward seems to be an attractive solution, there are some concerns and risks associated with this approach. At the agroecological scale, the location, landscape structure, climatic memory, and soil composition must be considered and can act as an environmental filter. This influence may have a strong negative impact on arable crops by the locally established relevant plant diseases and pathogenic microorganism communities in the rhizo- and phyllosphere of the plants. It can be assumed that certain areas are predestined for higher pathogen infestations, even in generally considered high-yield regions. Therefore, a holistic view of the ecological status of the region and the variability within a region on a smaller scale is essential.

Wheat, *Triticum aestivum* L., the most cultivated cereal in Europe, is particularly interesting due to its economic importance [11,12]. Fusarium head blight (FHB) in wheat, caused by a large group of *Fusarium* species, is already causing enormous economic damage and is under strict control, mainly due to the associated production of mycotoxins. Due to climate change, wheat susceptibility to FHB is expected to increase in predestined regions [13,14]. Another mycotoxin-producing phytopathogen is *Alternaria*, which also infests wheat and has an increased incidence in weakened plants [15,16,17]. Thus, to provide adequate crop protection and further select alternative cultivation sites, it is essential to study the influence of regional characteristics on the composition of the fungal community and its major pathogenic fungi.

Within-field distribution differences between *Fusarium* and *Alternaria* could already be detected [18]. These were mainly based on microclimate and showed that *Fusarium* was more abundant at humid and less warm sample sites. In contrast, *Alternaria* was more abundant at sparse sites and thus warmer and drier spots in the heterogeneous field [18]. Romero et al. [19] provide a clear summary of the influence of humidity and high temperature on fungal disease outbreaks and confirm that these two factors positively affect the occurrence and pathogen load of *Fusarium*.

Kivlin et al. [20] further showed that the assembly of the soil fungal community capable of aerial dispersal is mainly shaped by environmental filtering at the regional scale rather than by the range of its dispersal. Similarly, a study from Canada demonstrated that the population dynamics of *F. graminearum* are primarily directed by complex adaptive landscapes with varying regional selection pressures [21]. Nevertheless, no specific determinant could be identified. Instead, it appeared to be a result of the combination of climatic differences, site-specific undefined characteristics, and host prevalence at the various sites. In Europe, Pasquali et al. [22] contributed greatly to the coverage of *F. graminearum* and *F. culmorum* trichothecene genotypes. A database created through this work enables visualization apparent differences in the spatial distribution of the chemotypes of the respective *Fusarium* species.

However, sufficient studies on *Fusarium* species distribution in different regions and a list of site-specific characteristics shaping the *Fusarium* community are rare. For maize, a study by Goertz et al. [23], which investigated different *Fusarium* species throughout Germany and their mycotoxin formation, already recognizes regional differences. Precipitation and temperature differences were indicated as the main causes for the occurrence of different *Fusarium* species and mycotoxin profiles consisting of fumonisin, deoxynivalenol (DON), and zearalenone. Regional conditions such as soil and long-term mean precipitation were not further discussed. The impact of long-term mean precipitation values, in particular, could have a much stronger influence on the occurrence of *Fusarium* and an increase in the mycotoxin levels of DON than the amount of precipitation in the year of harvest [24]. Hence, it is hypothesized that the landscape forms a memory that affects the composition of microorganisms over a more extended period [24].

There are still insufficient studies on regional differences in the composition of the phyllosphere fungal community in Germany. Often only one species, such as *F. graminearum* or *F. culmorum*, was observed. Nevertheless, with the strong emergence of new molecular biological methods for community characterization, better insight into these complex structures is possible. Moreover, most metacommunity and metabarcoding studies in agriculture are related to soil [25,26]. Studies on the formation and composition of fungal communities in the phyllosphere of wheat are often lacking. Moreover, the few studies on mycotic phyllosphere composition usually focus on species composition and lose sight of the regional context [26,27,28]. Besides temperature and precipitation, no other parameters of the region are often considered.

This raises the question, on the one hand, to what extent the fungal community and, in particular, the *Fusarium* population in the phyllosphere of wheat differ in various regions. Do region-specific characteristics always result in the same dominant species, families, or fungal orders? On the other hand, can differences be detected only on a large scale between regions or on a small scale between fields in the same region? Further, to what can these differences be attributed? Answers to these questions are still lacking and are investigated in the present study.

For this purpose, we investigated plants of three regions (with 2–5 fields each) in North-East Germany with different wheat yield potentials, soil composition, and climatic conditions. We chose wheat after maize as the cropping system to favor the associated phytopathogenic fungal contamination with *Fusarium* and *Alternaria*. We hypothesized that all three regions would show different fungal community structures and dominant families. In addition, we expected that small-scale differences between fields in the same region, compared to regional differences, would be minor. We discuss typical regional characteristics as possible environmental filters, such as soil landscapes, soil values, yield potentials, long-term mean values of precipitation and temperature, and annual climate data. To characterize fungal infestations, wheat ears from conventionally managed fields were sampled and analyzed for *Fusarium* and *Alternaria* species composition and abundance, as well as the fungal community using a next-generation sequencing (NGS) approach targeting the nuclear ribosomal internal transcribed spacer (ITS) region as a universal DNA barcode marker for fungi. The purpose was to determine if relationships between the presence of *Fusarium*, *Alternaria*, and fungal communities and site characteristics can be observed.

## 2. Results

The results shown refer to ten study sites divided among the three regions Magdeburger Börde (MBB), Müncheberger Sander (MSA), and Uckermärkisches Hügelland (UKH). The regions differ in their yield potential and environmental parameters, such as soil type, temperature, and precipitation (Table 1).

### 2.1. Height of Wheat Plants

**Figure 1:**
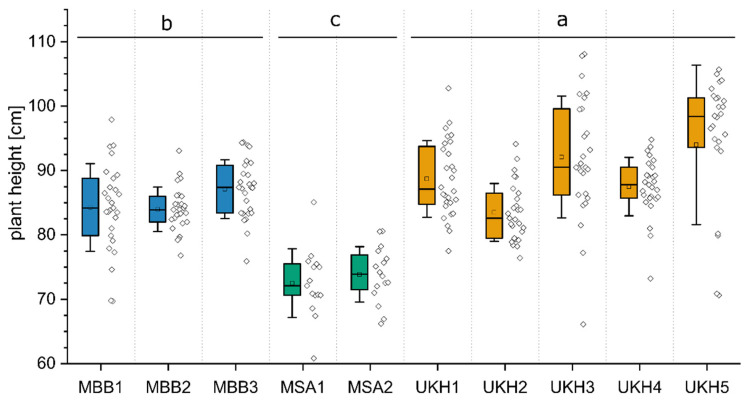
Boxplot of the plant heights of all experimental field sites. The corresponding individual data points are shown to the right. The box frames the 25% and 75% quantiles and represents both the median as a line and the mean as a square. The whiskers show a single standard deviation. MBB = Magdeburger Börde, MSA = Müncheberger Sander, UKH = Uckermärkisches Hügelland. All three regions are significantly different from each other at *p* < 0.001. Significance differences are represented by the letters a, b, and c.

The measured height of the plants can be related to biomass production. However, the results also depend on the variety and the use of growth regulators and, therefore, should be considered primarily in connection with other parameters. Comparing the mean values per field, field UKH5 had the highest values with a mean of 94 cm. In contrast, the two fields in the Müncheberger Sander region, MSA1 and MSA2, had the lowest heights, with means of 73 cm and 74 cm, respectively (Figure 1). All three regions are significantly different from each other. Thus, the observations on plant height confirm the ad hoc selection of the regions for their different yield potential.

### 2.2. Quantitative Abundances of Fusaria and Alternaria

**Figure 2:**
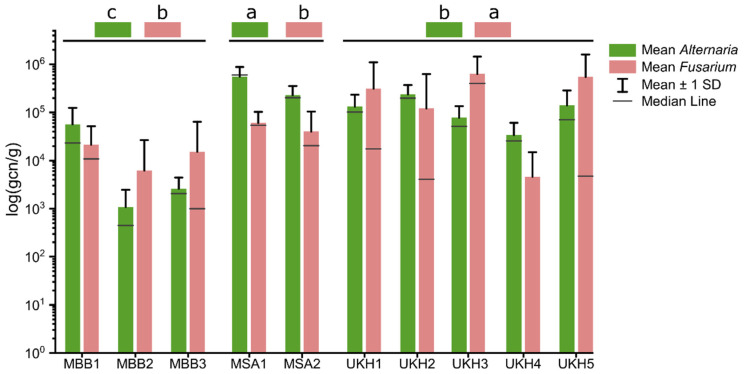
Logarithmized *Fusarium* and *Alternaria* abundances in wheat ears of the ten field sites analyzed by qPCR; gene copy number per gram dry mass (gcn/g). The whiskers show a single standard deviation. MBB = Magdeburger Börde, MSA = Müncheberger Sander, UKH = Uckermärkisches Hügelland. Significance differences are represented by the letters a, b, and c. *Alternaria* abundances differed significantly between all regions at *p* < 0.001. In terms of *Fusarium* abundances, UKH differed significantly from MSA at *p* < 0.01 and MBB at *p* < 0.001. *Fusarium* abundances between MBB and MSA were not significant. MBB had the lowest *Alternaria* and *Fusarium* levels of all three regions.

Quantitative analyses of wheat ears by qPCR confirmed *Fusarium* and *Alternaria* infestation in all fields. Nevertheless, *Fusarium* could not be found at all points. In UKH4 and MBB2, *Fusarium* could only be detected at 9 and 11 of 27 sampling points, respectively. Most Uckermark fields showed a *Fusarium* frequency above 5 × 10^4^ gene copy number per gram dry mass (gcn/g) (Figure 2). Only UKH4 deviated from this with 4.6 × 10^3^ gcn/g. The fields most affected by *Fusarium* were UKH1 (3.1 × 10^5^ gcn/g), UKH3 (6.3 × 10^5^ gcn/g), and UKH5 (5.5 × 10^5^ gcn/g). The fields from the Müncheberger Sander region had *Fusarium* frequencies of 5 × 10^4^ gcn/g. The fields from the Magdeburger Börde region were among those with the lowest *Fusarium* and *Alternaria* counts, such as the abundances we observed that was based on culture-dependent *Fusarium* isolation. Moreover, the MBB region is characterized by high soil quality and relatively lower long-term precipitation (Table 1). The field UKH4 (3.4 × 10^4^ gcn/g) also belonged to the fields with a comparatively low *Alternaria* abundance (Figure 2). *Alternaria* abundances in all regions differed significantly from each other. Fusaria abundance differed significantly between the MBB and UKH (α = 0.01) and MSA and UKH (α = 0.05) regions. In contrast, there were no significant differences in *Fusarium* abundances between the MBB and MSA regions (Figure 2).

### 2.3. Culture-Based Identification of Fusaria and Alternaria

Fusaria and alternaria were successfully isolated at all study sites. All species found were already associated with wheat in Germany. The goal of 20 isolates per species per field, however, was not met in most cases (Table A1). The following fields offered less than 20 *Alternaria* isolates: UKH2, UKH3, MSA1, MSA2, MBB1, and MBB2. The field UKH3 was the only one that reached the minimum of 20 *Fusarium* isolates. The sample site UKH4 stood out with a low isolation success of fewer than ten fusaria per field (Table A1) and reflected the generally low *Fusarium* abundance (Section 2.2).

**Figure 3:**
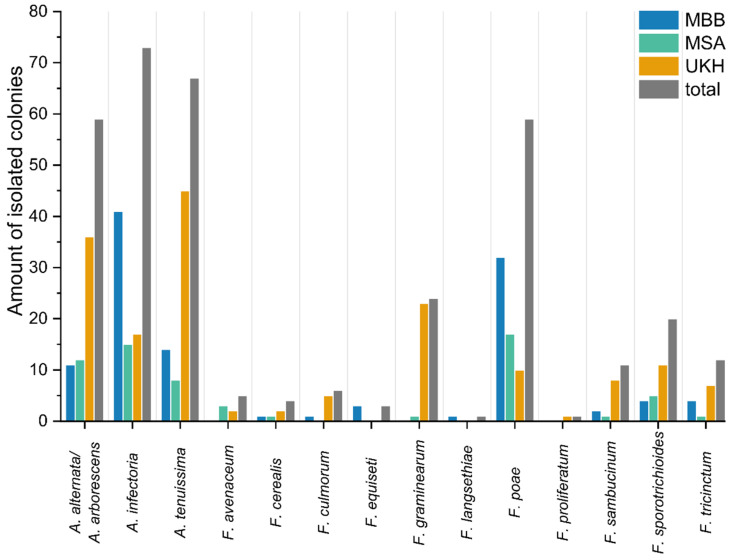
The total number of *Alternaria* and *Fusarium* isolated from wheat ears partitioned by species and region. MBB = Magdeburger Börde, MSA = Müncheberger Sander, UKH = Uckermärkisches Hügelland. MBB stood out with increased proportions of *A. infectoria* and *F. poae*. UKH was characterized mainly by *A. alternata/A. arborescens*, *A. tenuissima*, and *F. graminearum*. MSA had the lowest *Fusarium* species and exceeded UKH only with *F. poae*.

**Figure 4:**
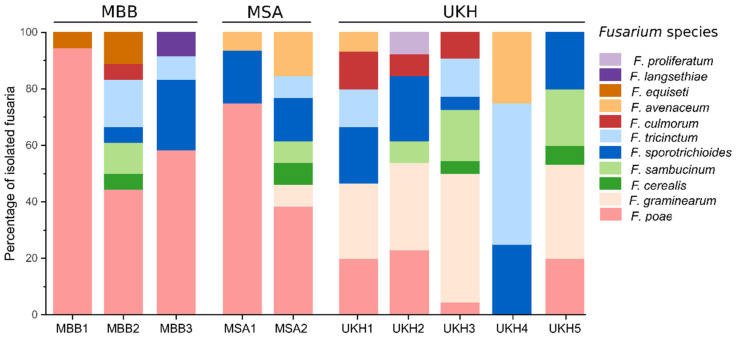
Bar chart showing the relative isolated fusaria species from wheat ears per field. MBB = Magdeburger Börde, MSA = Müncheberger Sander, UKH = Uckermärkisches Hügelland. Percentages of *Fusarium* isolates are grouped by testing sites. The total N per field can be found in Table A1. MSA and MBB were associated mainly with *F. poae,* while UKH was linked to *F. graminearum*.

The most abundant isolated *Fusarium* species were *F. poae*, *F. graminearum*, and *F. sporotrichioides* (Figure 3). The composition consisted of 2–7 *Fusarium* species per field, strongly distributed in a field-specific. In the Uckermark, field UKH4 stood out, resulting in only three different species. In addition, the species composition was unique, with a large proportion of *F. tricinctum* and *F. avenaceum*. *F. poae* was predominantly found in the fields outside the Uckermark. This was most evident in field MBB1, where 95% of the isolates were *F. poae*. In contrast, the fields in the Uckermark, except UKH4, had significantly more *F. graminearum* (20–30%, Figure 4), found only once in MSA2. Field UKH2 was the only one from which *F. proliferatum* and MBB3 from which *F. langsethiae* could be isolated (Figure 4). *F. equiseti* could only be found in the MBB region.

**Figure 5:**
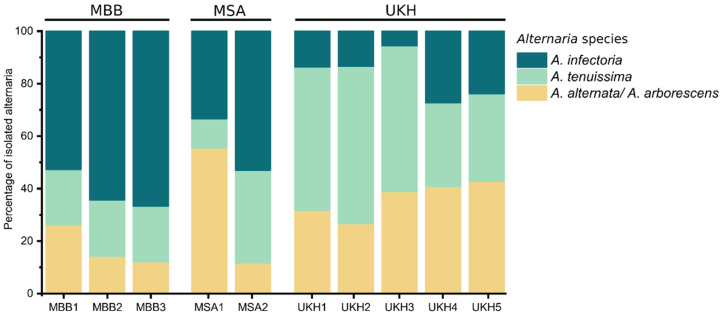
Bar chart showing the relative isolated alternaria species from wheat ears per field. MBB = Magdeburger Börde, MSA = Müncheberger Sander, UKH = Uckermärkisches Hügelland. Percentages of *Alternaria* isolates are grouped by testing sites. The total N per field can be found in Table A1. MSA and MBB were associated mainly with *A. infectoria,* while UKH was linked to *A. tenuissima* and a high percentage of *A. alternata*/*A. arborescens*.

Among the isolated species, *Alternaria* species groups were abundant in all fields. All three species groups had the lowest presence in MSA, a region with low soil quality and the highest amount of precipitation in the first half of 2020 compared to the other regions (Table 1). *A. infectoria* dominated in MBB, and UKH had twice as much *A. alternata*/*A. arborescens* and *A. tenuissima* as the other regions (Figure 3). There were no pronounced differences in *Alternaria* group composition between fields in a region, apart from MSA (Figure 5). In the fields of MBB, *A. infectoria* predominated. In contrast, in UKH, mycotoxin-producing alternaria accounted for 70% and more.

### 2.4. NGS Data Analysis (ITS)

After filtering out non-fungal sequences and excluding OTUs that occurred only two or fewer times per sample, 849 OTUs and 246 samples remained. These were affiliated with 5 phyla, 22 classes, 51 orders, 104 families, 104 genera, and 317 species. The average sequencing depth was 15,400 reads per sample. In total, the data comprised 3.8 million reads from 246 samples.

#### 2.4.1. Alpha Diversity

For alpha diversity analyses, the unfiltered fungal OTUs were rarefied to a uniform depth of 14,000 reads per sample, resulting in the removal of 119 samples and 142 OTUs. This was the level that removed problematic samples for alpha diversity but still left enough replicates for each landscape/field.

**Figure 6:**
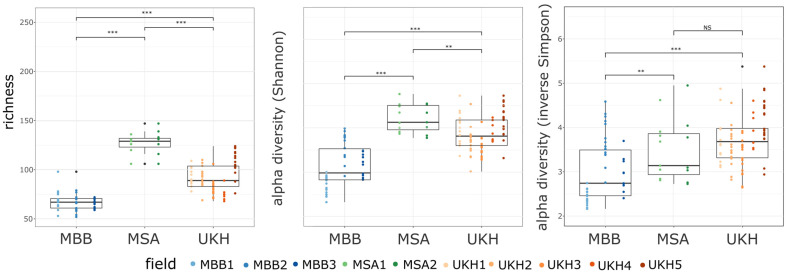
The differences in richness of OTUs (**left**) and alpha diversity (Shannon index: **middle**; inverse Simpson index: **right**) changes across landscapes and fields. Significance tested by pairwise Wilcoxon test and *p*-value adjusted by holm method. Significant at *** *p* < 0.001, ** *p* < 0.01, and NS = not significant. MBB = Magdeburger Börde, MSA = Müncheberger Sander, UKH = Uckermärkisches Hügelland. Significant differences in species richness and Shannon diversity prevailed between all regions. Inverted Simpson, giving less weight to rare species, affected mainly MSA and aligned the results with UKH without significance. Replicates per field: MBB1 = 17, MBB2 = 15, MBB = 10, MSA1 = 8, MSA2 = 7, UKH1 = 13, UKH2 = 16, UKH3 = 15, UKH4 = 7, UKH5 = 19.

Between the three sites, a significant difference existed in their alpha diversity. The Magdeburger Börde showed the lowest diversity and thus the lowest species diversity that could be recorded with the ITS primers (Figure 6). On the other hand, the fields in the Müncheberger Sander were doubled in richness compared to the Magdeburger Börde (Figure 6, left panel). The richness in the Uckermärkisches Hügelland also differed significantly from the other two sites and was in the middle range with its values. The same pattern was depicted by the Shannon alpha diversity index (Figure 6, middle panel).

In contrast, the UKH had the highest alpha diversity for the inverse Simpson index, giving less weight to the rare species (Figure 6, right panel) among landscapes, which was not significantly different from the MSA landscape. In terms of species richness, the MBB landscape had the lowest alpha diversity and was significantly different from the other landscapes studied. The field site MBB2 tended to have higher alpha diversity than 50% of this landscape data points (outliers box).

#### 2.4.2. Beta Diversity

**Figure 7:**
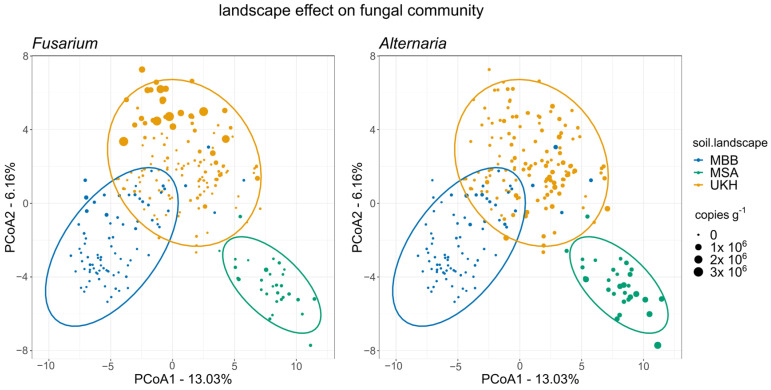
Principal coordinate analysis (PCoA) based on Aitchison distance of the relative fungal community composition affected by the landscape and quantitative abundance of *Fusarium* (**left**) or *Alternaria* species (**right**). Ellipses indicate 95% confidence intervals of a multivariate t-distribution. All three regions formed different clusters, with MSA entirely delineated from the others.

The field site explained 44% of the variance in community composition based on PERMANOVA analyses (Table A2). Principal coordinate analysis (PCoA) on relative fungal community composition revealed clear clusters for the three landscape areas. The Müncheberger Sander was highly separated and hardly overlapped with the other regions (Figure 7). The absolute abundance of *Fusarium* or *Alternaria* (Section 2.2) was driving the separation of fungal communities that belonged to the Uckermärkisches Hügelland or Magdeburger Börde, respectively.

**Figure 8:**
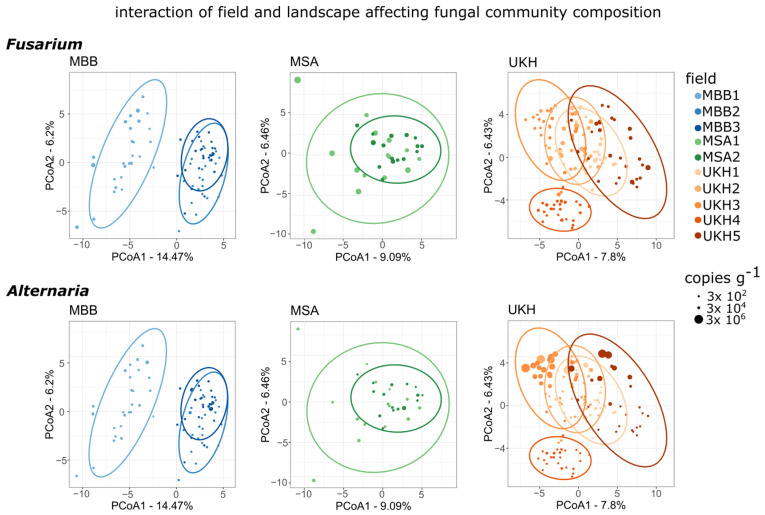
Principal coordinate analysis (PCoA) based on Aitchison distance of the relative fungal community composition interactively affected by landscape, field site, and quantitative abundance of *Fusarium* or *Alternaria* species (Section 2.2). Ellipses indicate 95% confidence intervals of a multivariate t-distribution. MBB = Magdeburger Börde, MSA = Müncheberger Sander, UKH = Uckermärkisches Hügelland. Within MBB and UKH, some fields were entirely distinct from their cluster (MBB1 and UKH4) and less overlapping (UKH3 and UKH5).

**Figure 13:**
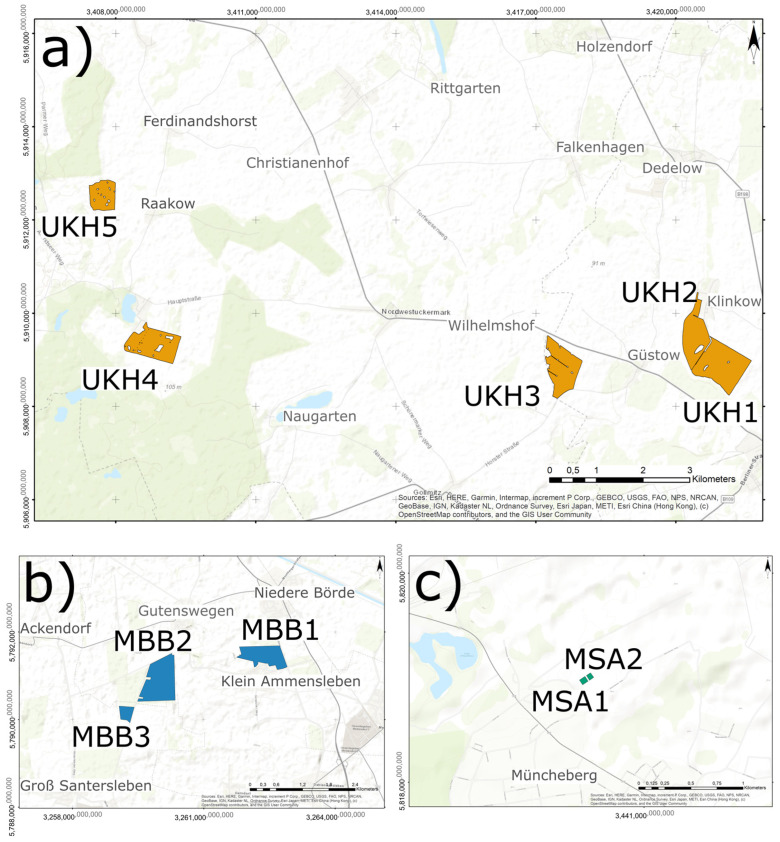
Field site position in the region (**a**) Uckermärkisches Hügelland (UKH), (**b**) Magdeburger Börde (MBB), and (**c**) Müncheberger Sander (MSA).

Within the landscape regions, there was a significant difference between the beta diversity of the fields, still explaining up to 16% of the total variance in the data (Table A3). PCoA further investigated the effect of individual fields within the landscapes on the fungal communities. Field MBB1 in the Magdeburger Börde formed its cluster, as did field UKH4 in the Uckermärkisches Hügelland. All other cluster study sites overlapped within the landscape region. Still, field UKH3 shared two of its sample points with UKH4. In addition, UKH3 and UKH5 exhibited a gradient characterized by the absolute abundance of *Alternaria* copies (Figure 8). The overlapping clusters also reflected the spatial situation of the fields very well. The fields MBB2 and MBB3 in the Magdeburger Börde, MSA1 and MSA2 in the Müncheberger Sander, and UKH1 to UKH3 in the Uckermärkisches Hügelland country were directly connected. Field MBB1, on the other hand, was about 2 km away from the other two fields. UKH4 was at least 2.5 to 13 km away from the fields in its region and was also surrounded by many forests (Figure 13).

#### 2.4.3. Taxonomic Composition

**Figure 9:**
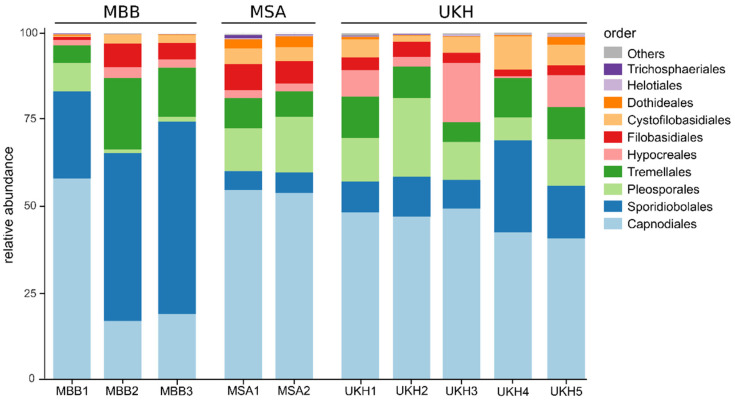
Bar chart showing the relative taxonomic composition of fungal order level in wheat ears averaged per field and region. MBB = Magdeburger Börde, MSA = Müncheberger Sander, UKH = Uckermärkisches Hügelland.

The relative abundances of OTUs were taxonomically aggregated at the order level for the examined fields (Figure 9). Data for *Fusarium* and *Alternaria* could not be compared beyond the genus level (Appendix A). The wheat ears studied were dominated mainly by species of the orders Capnodiales and Sporidiobolales. The third most abundant species belonged to the order Pleosporales, which includes *Alternaria*. The order Hypocreales, to which the fusaria belong, was the fifth most represented. Field UKH3 had a comparatively high proportion of Hypocreales, which was also reflected in the quantitative *Fusarium* abundances (Section 2.2).

**Figure 10:**
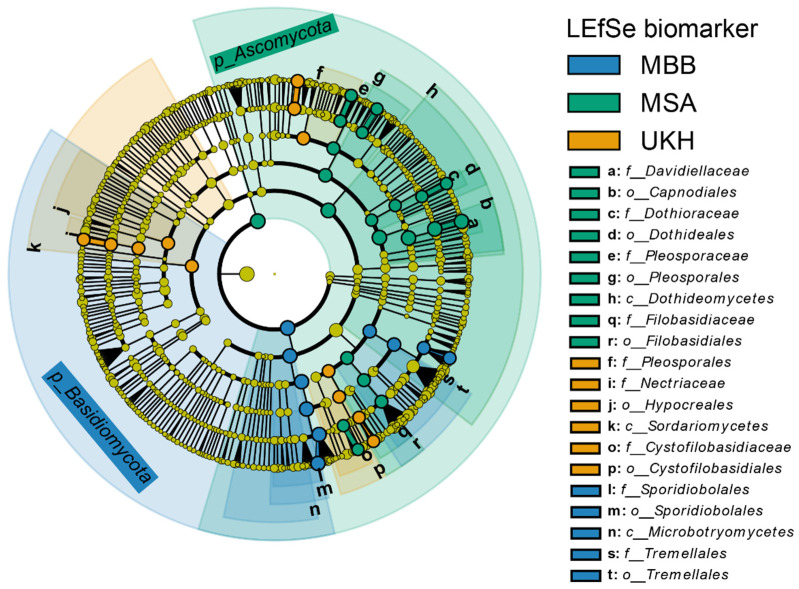
Linear discriminant analysis effect size (LEfSe) depicting differential abundant fungal taxa classified as biomarkers to the three regions analyzed (MBB = Magdeburger Börde, MSA = Müncheberger Sander, UKH = Uckermärkisches Hügelland), with node size scaled to relative abundance. Each region could be significantly assigned to a taxonomic order, which was used as the respective biomarker (MBB with Sporidiobolales, MSA with Capnodiales and Pleosporales, UKH with Hypocreales).

The order Hypocreales and its family Nectriaceae were consequently selected as a biomarker of the UKH region (Figure 10). The composition of the fields in MBB were comparable to each other, especially the two fields MBB2 and MBB3. Both had a substantially higher proportion of Sporidiobolales and Tremellales and a substantially lower proportion of Pleosporales, contrasting this region from the MSA and UKH region (Figure 9). Accordingly, Sporidiobolales was identified as a biomarker of the MBB region by the linear discriminant analysis effect size (LEfSe) method. The composition of field MBB1 was shifted by a relative increase in Capnodiales and Pleosporales, like the proportions of the fields from Müncheberg. Thereby, Capnodiales and Pleosporales were confirmed as a biomarker of the MSA region by the LEfSe analyses (Figure 10). In the Uckermark, UKH4 again differed from the fungal composition of the other and stood out, having a higher proportion of Sporidiobolales and Cystofilobasidiales within its region (Figure 9).

#### 2.4.4. Correlation of Fungal Community Composition with the Quantitative Abundance of *Fusarium* and *Alternaria* across and within Regions

**Figure 11:**
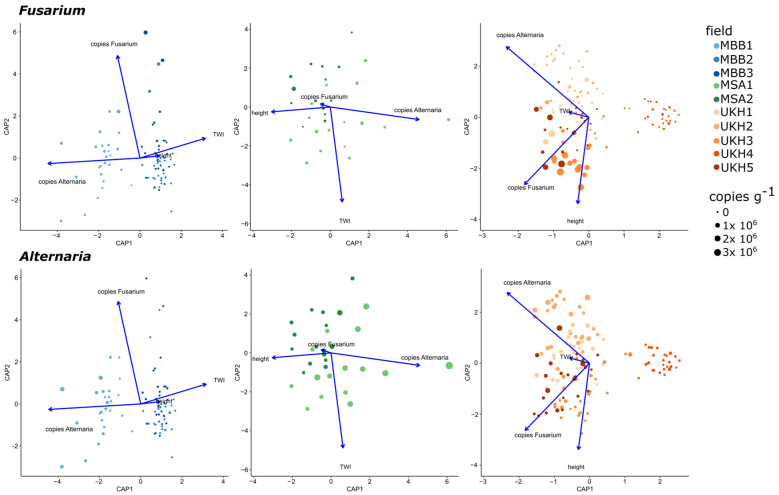
dbRDA plot indicating the effect of *Alternaria* and *Fusarium* abundances on the basis of qPCR, TWI, and plant height on the variation in the fungal community composition in the fields of the landscape regions. MBB = Magdeburger Börde, MSA = Müncheberger Sander, UKH = Uckermärkisches Hügelland.

**Figure A2:**
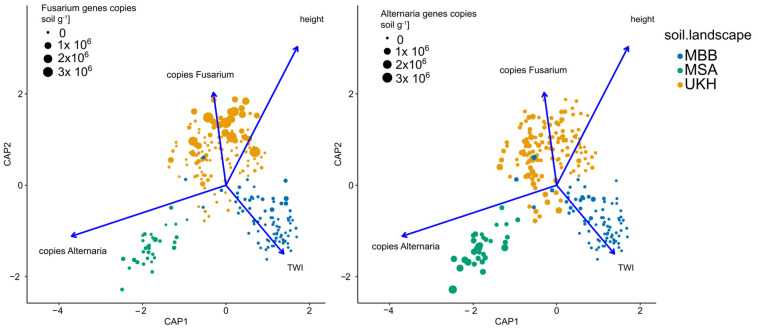
dbRDA plot showing the relations between fungal community and TWI, *Alternaria* and *Fusarium* abundances, and plant height that best explain the variation in the fungal community in the three landscape regions. MBB = Magdeburger Börde, MSA = Müncheberger Sander, UKH = Uckermärkisches Hügelland.

The relationships between fungal community and *Fusarium* and *Alternaria* abundances, as well as between plant height and soil moisture (represented by topographic wetness index; TWI), were visualized using a distance-based redundancy analysis (dbRDA; Figure 11. Across regions, the fungal community in UKH was significantly driven by the abundance of *Fusarium* quantified by qPCR, which was already suggested by exploratory analyses (Section 2.2). In contrast, the abundance of *Alternaria* in MSA had a stronger influence on the fungal community (Figure A2). No such correlation was evident in MBB. Plant height and TWI were weakly correlated with the fungal community (Table A5).

When comparing the fields of the MBB region, the fungal community in MBB1 correlated positively with *Alternaria* abundance and in MBB2 and MBB3 with TWI and only slightly with plant height (Figure 11, left panel). *Fusarium* abundance was positively correlated with the fungal community in all MBB fields. A strong negative correlation between plant height and *Alternaria* abundance was evident in MBB and MSA. Overall, the fungal community in MSA was similarly influenced by the height, TWI, and *Alternaria* abundance parameters presented in dbRDA (Figure 11, middle panel). *Fusarium* abundance had a minimal effect on the fungal community in MSA. In UKH, *Fusarium* abundances were correlated positively with plant height. The fungal community in UKH1–UKH3 and UKH5 was equally influenced by all parameters, with TWI exerting a weak influence. In contrast, field UKH4 remained unaffected by any parameter (Figure 11, right panel).

## 3. Discussion

### 3.1. Differences between and within Regions

All three regions not only differed in their abiotic characteristics but also appeared different in their microbial composition (Table 1). Different biomarkers (MBB: Sporidiobolales, MSA: Capnodiales, Pleosporales, UKH: Hypocreales) and the predominant *Fusarium* species (MBB and MSA: *F. poae*, UKH: *F. graminearum, F. tricinctum*) determined for every region made the variations visible. Furthermore, it was shown by MBB and UKH that a region could not inevitably be seen as a uniform entity. Thus, our results showed differences between regions and, in some cases, within the individual region. We could see that fields within a region often had the same characteristics the closer they were to each other. This context was reflected particularly clearly in the PCoA (Figure 8), where the adjacent fields MSA1 and MSA2, UKH1 and UKH2, and MBB2 and MBB3 overlapped considerably, if not entirely.

**Table 1:** Overview of all landscape features recorded for the regions Magdeburger Börde (MBB), Müncheberger Sander (MSA), and Uckermärkisches Hügelland (UKH) and the results obtained through this study.

Average Characteristics	MBB	MSA	UKH1–UKH3	UKH4–UKH5
Soil region	Old moraine landscapes	Young moraine landscapes	Young moraine landscapes	Young moraine landscapes
Soil type	Chernozem	Albeluvisol	Brown soils– para brown soils	Brown soils–para brown soils
SQR	≥85	<35–<50	50–<70	50–<70
Arable yield potential	very high	very low	medium	medium
TWI	8.3	7.8	7.8	7.0
Precipitation sum (mm) (Jan–July 2020)	277	291	233	262
Mean temperature (°C) (Jan–July 2020)	10.8	10.5	10.0	9.9
Height of wheat plants (cm)	85	73	88	91
qPCR fusaria (gcn/g)	14,220	50,256	353,505	275,063
qPCR alternaria (gcn/g)	19,997	391,773	149,091	87,041
Dominant *Fusarium*	*F. poae*	*F. poae*	*F. graminearum*	*F. graminearum,* *F. tricinctum*
Taxonomic biomarker	Sporidiobolales	Capnodiales, Pleosporales	Hypocreales	Hypocreales

#### 3.1.1. Magdeburger Börde (MBB)

MBB laying in old moraine landscapes is rated as a potentially high-yielding and reliable region and was able to stand out in our study with the lowest abundances for *Fusarium* and *Alternaria* (Table 1). In addition, the region is characterized by the lowest long-term average precipitation of the three regions. This parameter is hypothesized to be related to mycotoxin production by *Fusarium* and is thought to positively influence the active soil community, thus promoting *Fusarium* persistence [24].

According to the study by Müller et al. [24], a long-term mean precipitation value above 550 mm is associated with a 3.7-fold increase in mycotoxin levels in deoxynivalenol (DON) produced by *Fusarium*. Besides MBB (with 534 mm), the long-term precipitation mean was above the mentioned value of 550 mm (Table 1). *F. graminearum* and *F. culmorum* are the leading producers of DON [29,30] and occurred only once in MBB and MSA.

MBB1 was an exception in this region. While MBB2 and MBB3 were neighboring and had similar characteristics, MBB1 differed significantly in its taxonomic composition. MBB1 had more than twice as many Capnodiales and Pleosporales and was characterized by a 31-fold higher *Alternaria* abundance in the region.

All three fields differed in the isolated *Fusarium* species. From MBB1, only two species, *F. poae* and *F. equiseti,* could be isolated. MBB2 and MBB3 were similar in the *F. poae* proportion, but only in MBB2 could *F. cerealis*, *F. sambucinum,* and *F. culmorum* be isolated. In contrast, in MBB3, *F. langsethiae* and a significantly higher proportion of *F. sporotrichioides* than in MBB2 could be isolated. This result gives an idea of the variety of influences that ultimately affect community assembly and reflects the diversity within a region.

#### 3.1.2. Müncheberger Sander (MSA)

MSA is a region with meager yield potential and had the lowest wheat heights on average in our study. Furthermore, the region showed the highest *Alternaria* abundance, concluding in a high infection rate (Table 1). This is also reflected in the comparatively high representation of the order Pleosporales (includes *Alternaria*), which was statistically shown to be a biomarker for MSA. Low plant height may also indicate lower biomass and further a stressed plant. Due to the saprophytic nature of *Alternaria*, this phytopathogenic group increases when the plant and its defense mechanisms are weakened [18].

Furthermore, MSA showed the highest species richness compared to the three regions studied. Yet, the alpha diversity was only in the middle rank after excluding the rare species. An explanation may be found in the composition of the surrounding landscape. The landscape consisted of small patches of different arable crops, which formed a highly heterogeneous landscape. Some studies show the effects of smaller fields on the biodiversity of macroorganisms such as birds, plants, butterflies, bees, syrphids, carabids, and spiders [31,32]. These studies confirm that agricultural landscapes with smaller patches can improve biodiversity. Unfortunately, studies investigating this relationship at the microorganism level are still needed. Although there are studies on microbial biogeography [33,34], no conclusions can be drawn about microbial biodiversity in small-scale heterogeneous environments.

#### 3.1.3. Uckermärkisches Hügelland (UKH)

UKH was most affected by the mycotoxin-producing *Alternaria* species *A. alternata*/*A. arborescens* and *A. tenuissima*. In addition, there was a gradient in the abundance of *Alternaria* within the fields, particularly evident in UKH3 and UKH5. This could have been due to differences in microclimate or soil morphological changes on a small scale that we could not capture with our measure of abiotic values. On a smaller scale, for example, changes in microclimate could favor *Alternaria* at specific spots. For example, Schiro et al. [35] showed that alternaria were more abundant at warmer and drier sites within the field. UKH ranks in the midrange for many of our measurements for the three regions. UKH was most affected by *Fusarium* abundances and obtained the biomarker for the order Hypocreales, to which *Fusarium* also belongs. Two fields stood out in the region: UKH3 and UKH4.

UKH3 had with 6.3 × 10^5^ gcn/g the highest *Fusarium* abundances and was among the few fields from which more than 20 *Fusarium* isolates were retrieved. These results are also supported by the fact that the highest proportion of Hypocreales was found in UKH3 with respect to all field sites.

UKH4 contrasted with the lowest proportion of Hypocreales in the fungal composition and held a higher percentage of Sporidiobolales and Cystofilobasidiales. This pattern was also confirmed in *Fusarium* and *Alternaria* abundances, which appeared to be the lowest at all study sites. Four isolates composed of three species (*F. sporotrichioides*, *F. tricinctum*, and *F. avenaceum*) were obtained via the culture-dependent variant. There were three species, of which only *F. sporotrichioides* produces toxic trichothecenes, beauvericin, and fusarin C [36], while the other two species were insignificant in terms of mycotoxins. *F. graminearum*, the species predominant in all the other UKH fields, was not found in UKH4.

One explanation for the opposite results could be the landscape structure. In the eastern area of the UKH, the landscape is more homogeneous and becomes more heterogeneous in the western area. A particular attribute of UKH4 was the enclosure of the field by forests surrounded by three sides (Figure 13a). Forests can disrupt the exchange of organisms across the landscape and create a physical discontinuity between fields. Once again, there is a gap in studies examining the dispersal barriers of microorganisms, as the studies that have been conducted chiefly focus on animals and theoretical models [37,38].

However, the differences must always consider the fact that these were conventionally farmed fields, not always managed by the same farmer. This was an important factor that we could not consider in our analyses. The history of the fields, i.e., the long-term crop rotation of the field, also plays a crucial role in the microbial assemblage, as shown by Sommermann et al. [38] and Orrù et al. [39]. Studies also indicate that the combination of crop rotation, variety selection, and fungicide impact yield and the incidence of *Fusarium* [39,40,41]. In this regard, different practices can have different effects on different species. Drakopoulos et al. [41] have shown, for example, that reduced tillage practices can increase DON levels and the incidence of *F. graminearum*. On the other hand, *F. poae* responded to conventional tillage with an increased incidence.

### 3.2. Region-Specific Biomarkers and Their Microbiomes

Furthermore, it was noticeable that two dominant groups of *Fusarium* were found between the regions. In UKH, mainly *F. graminearum* was found, whereas *F. poae* predominated in the other two regions. Unfortunately, our study could not determine to what extent this was due to the type of soil tillage. *F. poae,* compared to *F. graminearum* and *F. culmorum,* is a rather mild pathogen capable of producing a wide variety of mycotoxins, including type A and B trichothecenes, beauvericin, and enniatin [42,43]. There has been a recent shift in the past 20 years in *F. graminearum* becoming the main *Fusarium* species in Europe, mainly due to the immense expansion of maize production on the continent [44]. Since *F. graminearum* also occurs on maize, crop rotations of wheat and maize create highly favorable conditions for this species. Nevertheless, *Fusarium* populations can shift considerably within and between cropping seasons [42,43]. Accordingly, the occurrence of a *Fusarium* species can fluctuate between regions and from one year to the following [42]. Therefore, multi-year studies would be required to confidently characterize MBB and MSA as a region dominated by *F. poae*.

Nevertheless, our study determined a specific phyllosphere ITS biomarker for each region. In other words, a fungal order is significant for this region and represents the main trait. The order Sporidiobolales, which predominates in MBB, originate from a wide range of habitats ranging from freshwater and marine ecosystems, soils, and plant tissues to Antarctic permafrost [45]. However, they have not yet been shown to have a positive effect on combating pathogens. Additionally represented prominently in MBB was the order Tremellales, with the genus *Cryptococcus*. This genus contains several strains of biological control agents, such as *C. flavescens*, *C. aureus*, and *C. carnescens*, which are extensively studied as biocontrol agents against *Fusarium* [46,47,48]. The extent to which the increased proportion of *Cryptococcus* in MBB decreased *Fusarium* abundance remains to be investigated.

In MSA, the two orders Capnodiales and Pleosporales were determined as biomarkers inhabiting several strains reducing disease severity. Several studies tested the order Capnodiales with the genus Cladosporium as a biocontrol agent [49]. In a field trial, Rojas et al. [49] detected a strong negative correlation between *Cladosporium herbarum* and three *F. graminearum* OTUs. *Alternaria* and *Phoma* are found in the order Pleosporales. Several species of both genera are known to act as antagonists against *Fusarium* [50,51,52]. For example, in a dual-culture plate test, *A. alternata* showed inhibitory activity against various *Fusarium* species, including *F. oxysporum*, *F. sporotrichioides*, *F. equiseti*, and *F. solani* [51]. In a climate chamber experiment, co-inoculation with *A. tenuissima* could limit the growth and mycotoxin production of *F. graminearum* [52]. Karlsson et al. (2021) also described that various *Alternaria* sp. and *Phoma* sp., with specific examples of *A. tenuissima* and *Phoma glomerata*, reduced disease severity caused by *F. graminearum*. Thus, the increased *Alternaria* abundances in MSA could contribute to the low *Fusarium* abundances.

The increased occurrence of *Fusarium* in UKH can also be found in the regional biomarker order Hypocreales. Even though the order also includes *Trichoderma*, which is used as a biocontrol agent [51,53,54], *Fusarium* did not seem to have been strongly affected by it during our sampling period.

### 3.3. Multifactorial Processes in Community Assembly

We could not establish significant correlations between regional abiotic factors and the established microbiomes. The results demonstrate that microbial assembly is a multifactorial process that is not yet fully understood. Regional conditions are only one factor contributing to this process. To understand the overall situation, other levels have to be taken into account, as differences between the regions were indeed found. As Kraft et al. [55] and Schlägel et al. [56] discussed, environmental filtering is only one piece of the community assembly puzzle; dispersal filtering and biotic filtering are also essential.

First, the dispersal capabilities of an organism must be sufficient to reach the site it intends to colonize [56]. There is limited information in the literature on the wind dispersal of different *Fusarium* species. In a wind tunnel experiment, *Fusarium* spores of different species exhibited different wind dispersal tendencies and indicated probable wind dispersal with carrier media or mobile linkers [57]. Kivlin et al. [20] showed that dispersal was not the limiting filter for regional fungal composition in soil. Instead, it suggested that site-specific abiotic factors, i.e., environmental filters, in combination with stochastic or neutral processes, were responsible for regional composition.

Secondly, for survival and successful reproduction, the abiotic environmental conditions in the new site must be suitable [56]. For example, Kivlin et al. [20] found that soil fungal community composition correlated with soil nitrate, ammonium, soil C:N, and soil moisture. Thapa and Prasanna [58] described microbial diversity in the phyllosphere as a reflection of environmental conditions. Abiotic factors, such as season, water content, relative humidity, soil composition [58], geographic location, solar radiation, pollution, and nutrients [59], can affect the structure and abundance of the phyllosphere community.

Third, some biotic drivers challenge persistence in the presence of other species [56]. On the one hand, the genetic network of the plant can influence the microbiome, as Shakir et al. [25] summarized. On the other hand, local competition takes place on the plant surface. There are two broad groups, pathogens and beneficials, which can act either directly against the pathogens or indirectly through the plant [26,50].

The complexity behind the filter processes in the composition of a community is high and far from being well understood. Future studies should try to choose a multifactorial approach and serve as many filter scales as possible. In addition, a multi-year experimental design is advisable to capture fluctuations in the occurrence of some species.

### 3.4. Closing Remarks

With regional differences in mind, we created the first detailed study in Germany that examines the fungal phyllosphere of wheat. Our results show apparent differences, although their final explanation occasionally remains open. The importance of the phyllosphere gradually gains interest. However, the importance is not less valuable than the rhizosphere and soil community, especially with increasing diseases of the above-ground plant parts such as the re-emerging rust fungi *Puccinia graminis* [60].

The results can serve as a guide for the shifting of cultivation regions, which should also be chosen on the basis of their phytopathogenic infection potential in the future. In this regard, special consideration should be given to the region-specific potential as an environmental filter. We also hope that the study will inspire the inclusion of microbial metacommunity analyses in the selection of future growing regions. As a result, region-specific characteristics will be better understood and can be applied in evaluating the agricultural suitability of new regions.

## 4. Materials and Methods

### 4.1. Study Sites and Sample Collection

**Figure 12:**
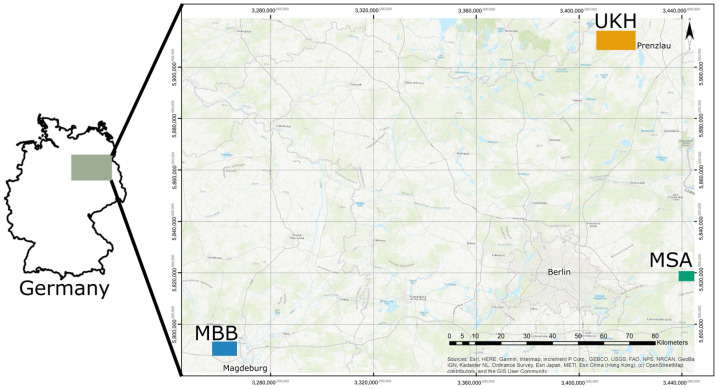
Map of study sites in North-East Germany in the region Uckermärkisches Hügelland (UKH) with five fields, Müncheberger Sander (MSA) with two fields, and Magdeburger Börde (MBB) with three fields.

The study sites were in North-East Germany (Figure 12) and sampled in 2020. Only wheat fields with maize as a previous crop were selected to increase the incidence of pathogens. The cultivation and treatment of the crops took place according to the practices of conventional agriculture. All farmers practiced reduced tillage. The selection included fields that were close to each other as well as fields that were at different distances from each other (from 20 m to 196 km) (Table 2).

The study area was selected primarily because of its different wheat yield potential, including fields from the regions Magdeburger Börde (MBB), Müncheberger Sander (MSA), and Uckermärkisches Hügelland (UKH) (Figure 12). Ten field sites were numbered according to the region in which they lay (Figure 13).

### 4.2. Landscape Characteristics

#### 4.2.1. Soil and Yield Potential

Characteristic properties of soil composition and site-specific yield potential were determined from the soil atlas of the Federal Institute for Geosciences and Natural Resources using the Müncheberg Soil Quality Index (SQR) (https://geoviewer.bgr.de/mapapps4/resources/apps/bodenalas/idex.html?lang=de&tab=boedenDeutschlands (accessed on 23 August 2022)). We used the ArcMap software (v 10.7.0.10450, Esri, Redlands, CA, USA) for the landscape mapping and the calculation of the topographic wetness index (TWI). The TWI describes the tendency of an area to accumulate water and is, therefore, often used as an indicator for soil moisture [61]. The function for TWI was developed by Beven et al. [62], with TWI being defined as ln(α/tan β), where α (α = A/L) is the local upslope area (A) draining through a certain point per contour length (L) and tan β is the local slope. A grid width of 20 m was used to calculate the TWI with the ArcMap Toolbox “Spatial Analyst”.

The landscape Magdeburger Börde is located in the soil region old moraine landscape and consists of soils of the Loess Uplands with the predominant soil type Chernozem. In the SQR, all three fields receive a value of ≥85, indicating a very high arable yield potential. The TWI ranges from 7.5 to 9.2, showing a slight increase in water accumulation compared to the other regions.

The landscape Müncheberger Sander ”elon’s to the soil region young moraine landscape. It is mainly characterized by soils of the outwash plain and dry valley sands as well as the sandy plates and sandy terminal moraine. The main component of the soil type is Albeluvisol. MS1 and MS2 fields received an extremely low to very low SQR (value: from <35 to <50) and therefore represent a region with very low arable yield potential. The two fields in Müncheberg showed a TWI of 8.3 (MSA1) and 7.3 (MSA2) and thus an average TWI for this region of 7.8.

The soil in the landscape region Uckermärkisches Hügelland belongs to the soil region young moraine landscape and is characterized by soils of the ground moraine plates and loamy end moraines. Soil types such as brown and para brown soils mainly characterize this landscape. Our experimental design classified it as low to middle in the SQR (50 to <70) and represents a region with medium yield potential. The TWI ranges from 6.9 to 8.1 depending on the field position; with an average value of 7.5, the TWI in this region is the lowest compared to the other two. Thus, in this region is relatively less water accumulation.

#### 4.2.2. Climate

The Climate data in 2020 were obtained from the website of the German Meteorological Service (https://www.dwd.de/DE/leistungen/cdc /cdc_ueberblick-klimadaten.html (accessed on 23 August 2022)). The meteorological data from January to July 2020 were summarized in Table 3. The long-term average temperature and precipitation of each region are presented in Table 4. Given the spatial distance of the fields in UKH, data from two weather stations, near UKH1–UKH3 and UKH4–UKH5, were listed as references.

### 4.3. Sample Collection

The field samples were collected in 2020, approximately two weeks after flowering and three weeks before harvest. Every field had 27 sample points (except fields MSA1 and MSA2 with 15 and 16 sample points, respectively) and three to six transects at approximately 25 m distance between each other to cover a broader range in the field and to balance heterogeneous characteristics within a field. Within a transect, there were up to nine sampling points at 50 m apart. The total number of collected field samples was 247. At each sampling point, the height of 10 wheat plants in a one-square-meter segment was measured to provide an indirect value for site productivity. Fifteen wheat ears were cut at each sampling point.

### 4.4. Fungi Isolation and Determination

For each sampling point, ten kernels of the freshly harvested wheat ears were placed on Petri dishes with potato dextrose agar (PDA; Merck, Heidelberg, Germany) supplemented with chloramphenicol (CA); i.e., with five kernels per dish. The Petri dishes were incubated at 24 °C for two days in darkness and afterward placed in a box with a UV-A lamp (wavelength 320–400 nm) under a UV-A light/darkness automatic cycle (12 h UV light, 12 h darkness) in room temperature for at least four days to support sporulation and typical development of the mycelium color. From the PDA + CA Petri dishes, we attempted to collect 20 *Fusarium* and 20 *Alternaria* fungi of as many species groups for each field to overview the fungal diversity available on the selected wheat fields. The selected colonies were transferred to fresh PDA and synthetic nutrient agar (SNA, [63]) plates for further species determination on the basis of microscopic and macroscopic characteristics described by Leslie and Summerell [64].

Single-spore isolates of *Fusarium* were generated in preparation for the subsequent sequencing following the protocol of Noman et al. [65]. The mycelium on the new PDA plates was again left to grow for three days in the incubator and afterward for seven days in the UV box and stored at 7 °C to be used for further procedures.

A representative isolate selection of all *Fusarium* species was sent for sequencing to confirm the accuracy of the *Fusarium* species determined by the culture-dependent method. Therefore, DNA was extracted from half of the mycelium of an overgrown plate according to a customized standard protocol of the NucleoSpin^®^ Soil Kit (Macherey-Nagel GmbH and Co. KG, Düren, Germany). The lysis step from the standard protocol was modified in that the lysate was left at room temperature for 1 h before spinning down. In addition, the samples were centrifuged at 13,000 rpm instead of 11,000 rpm in all steps. PCR amplification and amplicon sequencing were performed by LGC Genomics (Berlin, Germany). For a species-specific *Fusarium* determination, the region of the translation elongation factor 1-alpha (TEF) was selected according to the protocol of the *Fusarium*-ID project [66]. The following primer pair was used for sequencing the TEF region: ef1 forward primer 5′-ATGGGTAAGGA(A/G)GACAAGAC-3′, ef2 reverse primer 5′-GGA(G/A)GTACCAGT(G/C)AT CATGTT-3′. To confirm the preliminarily identified isolates of *Fusarium* species, we selected the FUSARIUM-ID database accessible via the Internet (http://isolate.fusariumdb.org (accessed on 27 January 2022)), whose protocol we also followed for PCR amplification. On the platform, we used the integrated nucleotide BLAST query to identify *Fusarium* species, accessing over 5558 reference sequences from 1844 isolates representing more than 200 phylogenetically distinct species. The BLAST program provided results in terms of best matches with the sequences available in the database. The results were compared with the previously determined microbiological data and revised. Thereby, we gave higher importance to the data from sequencing.

### 4.5. Fungal Quantification with qPCR

Fifteen wheat ears were collected from each sampling point, then dried at 60 °C for 48 h and ground with a disk mill RS200 (Retsch, Haan, Germany) at 1000 rpm for 1.15 min. From the ground material, 50 mg were taken for genomic DNA extraction using the DNeasy Plant Mini Kit (QIAGEN GmbH, Hilden, Germany). For a detailed description of the method, see Müller et al. [67]. Quantifying *Fusarium* (efficiency > 0.89 and R2 > 0.96) and *Alternaria* (efficiency > 0.91 and R2 > 0.998) by qPCR was based on the probes and primers listed in Table 5 and was previously described in detail by Gerling et al. [68]. All qPCR assays included negative controls and were run in duplicate. The fungal strains used to generate the standard curves were obtained from a culture collection of fungi at the Leibniz Centre for Agricultural Landscape Research Müncheberg.

### 4.6. NGS

The extracted DNA from the qPCR analysis was used for next-generation sequencing (NGS) to examine the entire fungal composition. Therefore, the first nuclear ribosomal internal transcribed spacer region (ITS1) used the ITS1F (5′-CTTGGTCATTTAGAGGAAGTAA-3′) and ITS2 (5′-GCTGCG TTCTTCATCGATGC-3′) fungal specific primer pair [69]. PCR amplification and amplicon sequencing using an Illumina MiSeq platform were performed by LGC Genomics (Berlin, Germany).

### 4.7. Data Management

Research data are available at the ZALF data storage: https://doi.org/10.4228/zalf-m1vb-v194 (accessed on 13 December 2022), and raw sequencing data at the Sequence Read Archive (SRA) of NCBI: https://www.ncbi.nlm.nih.gov/sra/PRJNA876280 (accessed on 1 October 2022). The sequence data that support the identification of *Fusarium* species of this study are available from the corresponding author (A.H.), upon reasonable request.

### 4.8. Statistical Analysis

Plant height, fungal isolation, and qPCR data were statistically analyzed and presented using OriginPro (version 2019b; OriginLab Corporation, Northampton, MA, USA). Data were tested for normal distribution (Kolmogorov–Smirnov test) and homogeneity of variances (Levene test). Differences between the different fields and the three regions were analyzed with the one-way ANOVA and the Bonferroni post hoc test (α = 0.001).

The basic bioinformatics pipeline for the NGS data were performed by LGC Genomics (Berlin, Germany). According to the company data description, the procedure was as follows: The raw read quality was assessed with FastQC v0.11.9, and demultiplexing was performed using bcl2fastq, followed by merging forward and reversed reads using BBMerge v34.48. Pre-processing and OTU picking from amplicons was performed with Mothur v1.35.1: filtering of short products elimination of chimera with the uchime algorithm. CD-HIT-EST v4.6.1 clustering was performed at the 97% identity level, with cluster representative sequence re-selection to the most abundant sequence instead of the default representative sequence (default: longest sequence). Taxonomic classification of OTUs was performed using the UNITE version 6 reference database and RDP tool (11.4). Species-level resolution of *Fusarium* and *Alternaria* was insufficient for most of the hits we obtained, and thus the evaluation was made at the genus level.

Data obtained from the sequencing company were imported into the R statistical environment, and non-fungal OTUs were discarded. For alpha diversity analyses, the unfiltered fungal OTUs were rarefied to a uniform depth of 14,000 reads per sample, resulting in the removal of 119 samples and 142 OTUs. This was the level that removed problematic samples for alpha diversity but still left enough replicates for each landscape/field. Moreover, for the beta diversity analyses, only OTUs occurring at least two times per sample were recovered. For beta diversity, we used Hellinger transformation, relative abundances, and normalization/transformation included in the LEfSe tool.

Diversity analyses were based on phyloseq [70] and utilized robust Aitchison distance implemented by vegan::vegdist [71] and further made use of ape::pcoa [72] for unconstrained or capscale function for constrained or unconstrained ordination and vegan::adonis2 [73] for permutational analyses of variance (PERMANOVA). Assumptions of PERMANOVA were checked using betadisper and permutest. Moreover, ggplots2 [73] and microeco [74] were used to produce visualizations. Biomarker taxa that were differential abundant across analyzed regions and can reflect their biological conditions were identified using LEfSe [75], defining an LDA score of 4 as a cutoff.

## Figures and Tables

**Table 2:** Distance matrix measured from field border to field border in kilometers for the sampled fields. MBB = Magdeburger Börde, MSA = Müncheberger Sander, UKH = Uckermärkisches Hügelland.

Field	MBB1	MBB2	MBB3	MSA1	MSA2	UKH1	UKH2	UKH3	UKH4	UKH5
MBB1	0									
MBB2	1.4	0								
MBB3	2.6	0.2	0							
MSA1	179.7	182.2	183.2	0						
MSA2	179.7	182.2	183.3	0.02	0					
UKH1	196.3	198.4	199.8	91.3	91.3	0				
UKH2	196.3	198.4	199.9	92.0	92.0	0	0			
UKH3	193.6	195.7	197.1	92.1	92.1	2.4	21.8	0		
UKH4	187.0	189.0	190.5	95.2	95.2	11.0	10.7	7.8	0	
UKH5	188.4	190.4	191.8	98.7	98.7	12.8	12.4	9.7	2.5	0

**Table 3:** Meteorological data from January to July of 2020 for the regions Magdeburger Börde (MBB), Müncheberger Sander (MSA), and Uckermärkisches Hügelland (UKH). Air temperature at 2 m high, relative humidity, and precipitation sum were collected. Data were obtained from Deutscher Wetterdienst raster maps (https://www.dwd.de/DE/leistungen/cdc/cdc_ueberblick-klimadaten.html (accessed on 23 August 2022)).

MBB	January	February	March	April	May	June	July
Ø air temperature (°C)	4.5	6.2	5.8	10.7	12	18.4	18.1
Ø rel. humidity (%)	83.1	76.2	66.5	53.4	61.7	65.2	64.9
precipitation sum (mm)	23	81	27	7	27	72	40
MSA							
Ø air temperature (°C)	4.0	5.7	4.9	9.9	12.6	18.5	18.1
Ø rel. humidity (%)	84.0	78.1	69.3	55.9	-	67.9	68.2
precipitation sum (mm)	42	77	24	17	29	59	43
UKH1–UKH3							
Ø air temperature (°C)	4.1	5.3	4.5	8.9	12.6	17.4	17.4
Ø rel. humidity (%)	89.2	81.2	74.8	63.3	70.5	72.0	68.7
precipitation sum (mm)	36	53	36	14	27	37	30
UKH4–UKH5							
Ø air temperature (°C)	4.0	5.2	4.4	8.9	12.4	17.3	17.0
Ø rel. humidity (%)	89.2	81.2	74.8	63.3	70.5	72.0	68.7
precipitation sum (mm)	40	56	38	13	26	52	37

**Table 4:** Long-term average temperature and precipitation data from 1991 for the regions Magdeburger Börde (MBB), Müncheberger Sander (MSA), and Uckermärkisches Hügelland (UKH) were summarized. Data obtained from Deutscher Wetterdienst raster maps (https://www.dwd.de/DE/leistungen/cdc/cdc_ueberblick-klimadaten.html (accessed on 23 August 2022)).

Region	Long-Term Average Temperature (°C)	Long-Term Average Precipitation (mm)
MBB	9.9	534
MSA	9.5	553
UKH1–UKH3	9.1	553
UKH4–UKH5	9.0	580

**Table 5:** Primers for quantitative identification of *Fusarium* and *Alternaria* via qPCR.

S SUF pl3	Probe	5′-ACCCTTACCGAGCTCAGCGGCTTCCTATT-3′
Fa PL3 f	forward	5′-TACCCCGCCACTCGAGCG-3′
Fus pl rev	reverse	5′-TTGAGCTTGTCAAGAACCCAGGCG-3′
Alt-Prt	probe	5′-TGGGTTCGCCCACCACTAGGACA-3′
Alt-F	forward	5′-TCTTTTGCGTACTTCTTGTTTCCTT-3′
Alt-R	reverse	5′-TTACTGACGCTGATTGCAATTACA-3′

**Figure A1:**
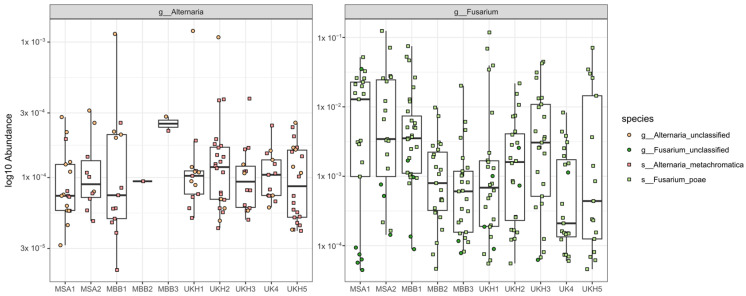
Boxplot showing the taxonomic composition of fungal species for *Fusarium* and *Alternaria* per field and region. MBB = Magdeburger Börde, MSA = Müncheberger Sander, UKH = Uckermärkisches Hügelland.

## Appendix A

**Table A1:** Summary of the isolated fusaria and alternaria from wheat ears at the different sample sites. The target was to obtain 20 isolates per field and genus. MBB = Magdeburger Börde, MSA = Müncheberger Sander, UKH = Uckermärkisches Hügelland.

Field	Isolated Colonies		Total
	*Alternaria*	*Fusarium*	
MBB1	19	18	37
MBB2	14	18	32
MBB3	33	12	45
MSA1	18	16	34
MSA2	17	13	30
UKH1	22	15	37
UKH2	15	13	28
UKH3	18	22	40
UKH4	22	4	26
UKH5	21	15	36
total	360	260	620

**Table A2:** The effect of landscape on fungal community composition determined by PERMANOVA on Aitchison distances.

	Df	SumOfSqs	R2	F	Pr (>F)	
Soil landscape	2	9.2999	0.43751	203.64	0.001	***
Field	7	6.5676	0.30897	41.09	0.001	***
Residual	236	5.3887	0.25351			
Total	245	21.2562	1			

Signif. code: ‘***’ 0.001. Formula: fungal distance matrix ~ soil. landscape + field. *** Terms added sequentially (first to last); permutation: free; number of permutations: 999.

**Table A3:** The effect of field site on fungal community composition determined by PERMANOVA on Aitchison distances.

	Df	SumOfSqs	R2	F	Pr (>F)	
MSA						
Field	2	1384	0.14592	6.5776	0.001	***
Residual	77	8100.8	0.85408			
Total	79	9484.8	1			
MBB						
Field	1	206.7	0.04366	1.3238	0.01	**
Residual	29	4528.3	0.95634			
Total	30	4735	1			
UKH						
Field	4	3437.3	0.16316	6.3368	0.001	***
Residual	130	17,629.3	0.83684			
Total	134	21,066.6	1			

Signif. codes: ‘***’ 0.001 ‘**’ 0.01. ** Formula: fungal distance matrix ~ field. *** Terms added sequentially (first to last); permutation: free; number of permutations: 999.

**Table A4:** The effect of TWI, *Alternaria* and *Fusarium* abundances, plant height, and SQR on fungal community composition determined by permutation test for capscale under reduced model on Aitchison distances. Related parameters to dbRDA plot (Figure 11).

	Df	Variance	F	Pr (>F)	
Model	8	37.083	83.491	0.001	***
Residual	236	131.025			
Terms					
TWI	1	2.903	52.281	0.001	***
qPCR *Allternaria*	1	10.266	184.912	0.001	***
qPCR *Fusarium*	1	3.287	59.197	0.001	***
Plant height	1	2.474	4.457	0.001	***
SQR.rating	4	18.153	81.743	0.001	***
Residual	236	131.025			
Correlation with axis					
	CAP1	CAP2			
TWI	0.25268324	−0.2731339			
copiesALT	−0.67589029	−0.2029782			
copiesFUS	−0.05474777	0.3695871			
Height	0.31313261	0.5519229			

Signif. codes: ‘***’ 0.001.Formula: Aitchison distance ~ TWI + copiesALT + copiesFUS + height + SQR.rating. *** Terms added sequentially (first to last); permutation: free; number of permutations: 999.

**Table A5:** Correlation between fungal community and TWI, *Alternaria* and *Fusarium* abundances, and plant height. Related parameters to dbRDA plot (Figure 11).

Variables	Correlation Method	Correlation Coefficient	*p*-Value	*p*-Adjusted	
Height	Pearson	0.06262916	0.023	0.023	*
TWI	Pearson	0.13793507	0.001	0.001333	******
copiesALT	Pearson	0.13893371	0.001	0.001333	**
copiesFUS	Pearson	0.18767443	0.001	0.001333	**

Signif. codes: ‘**’ 0.01, ‘*’ 0.5.
